# Borderline Myxoid Adrenocortical Neoplasm: A Case Report

**DOI:** 10.7759/cureus.110972

**Published:** 2026-06-16

**Authors:** Maria-Anthi Chantzi, Theodoros Sidiropoulos, Spyridon Christodoulou, Vaia Stafyla, Efthymios Poulios

**Affiliations:** 1 School of Medicine, National and Kapodistrian University of Athens, Athens, GRC; 2 Fourth Department of Surgery, Attikon University Hospital, Athens, GRC

**Keywords:** adrenal gland, borderline malignant behavior, hypertension due to neoplasm, myxoid variant, neoplasm of the adrenal cortex, retroperitoneoscopic adrenalectomy

## Abstract

Myxoid adrenocortical neoplasm (MAN) is an exceptionally rare variant of adrenal tumor arising from the cortex, with limited cases reported globally. A minor proportion of these are classified as having borderline malignant potential (BMAN). Typically, these neoplasms exhibit a biphasic histological pattern, with both myxoid and conventional non-myxoid regions. Their identification remains challenging, particularly when the myxoid component predominates. The presence of extensive myxoid stroma can obscure traditional histological criteria crucial for malignancy assessment, such as those outlined in the Weiss criteria, thus complicating risk stratification. We report the case of a 34-year-old female patient who presented to the outpatient clinic with a three-month history of persistent headaches, episodic hypertension, and fatigue. Her blood pressure fluctuated between 140-200 and 90-110 mmHg. Imaging revealed an incidental adrenal mass. No significant past medical history or family history of hypertension or cardiovascular disease was reported. The patient subsequently underwent a posterior retroperitoneoscopic adrenalectomy of the left adrenal gland. Histopathological analysis revealed a tumor with prominent myxoid features and a low Weiss score (2), without evidence of capsular or periadrenal invasion, confirming the diagnosis of BMAN. The rarity and distinct histomorphological features of BMANs complicate their diagnosis. This case contributes to the limited literature and underscores the diagnostic challenges involved. The inclusion of these tumors in the differential diagnosis of adrenal masses, particularly when related hormonal abnormalities are present, as well as thorough clinical and histopathological evaluation, is crucial. The favorable outcome of the patient highlights the significance of early diagnosis and complete surgical excision. However, further research is essential for understanding the biological behavior of these neoplasms and for refining the diagnostic criteria and management strategies.

## Introduction

Adrenal cortex tumors comprise a broad spectrum, ranging from benign adrenocortical adenomas to adrenocortical carcinomas, with the latter being among the most aggressive endocrine malignancies [1,2]. These tumors are often discovered incidentally during imaging performed for unrelated reasons and present a wide range of clinical manifestations. They may present as non-functioning, or with hormone excess, most commonly involving cortisol, aldosterone, or, less frequently, adrenal androgens, leading to endocrine syndromes such as Cushing syndrome or primary hyperaldosteronism. While most adrenocortical adenomas are benign and clinically inactive, a minority may demonstrate endocrine activity or malignant potential, necessitating careful histopathological evaluation and risk stratification. Understanding this clinical spectrum is essential for interpreting uncommon variants, such as myxoid adrenocortical neoplasms (MANs), which may not follow typical morphological patterns [1,3,4].

MANs constitute a rare histologic variant within this spectrum. These tumors are characterized by a prominent myxoid stromal matrix, interspersed with tumor cells of adrenocortical origin [1,4]. To date, fewer than a hundred cases have been reported globally [5,6]. MANs were first reported in the literature by Tang et al. in 1979 [4] and subsequently clearly defined in 2000 [1]. This variant has been described in both adenomas and carcinomas; however, most reported cases have exhibited malignant or at least borderline malignant features, with the myxoid component representing 10-95 % of the tumor volume [1,4].

Histological identification of these tumors appears challenging [1,4,7]. The extensive myxoid stroma can obscure traditional architectural and cytologic features used in established malignancy criteria, such as the Weiss scoring system. Therefore, despite the Weiss score’s utility in diagnosing conventional adrenocortical tumors, its relevance in myxoid neoplasms is limited [3]. It is underscored in the existing literature that the diagnostic and prognostic diversity of adrenocortical neoplasms necessitates the integration of histologic, immunohistochemical, and molecular findings in their evaluation to prevent under-recognition or misclassification [3,7].

Given the limited number of published reports, each additional case may contribute to understanding the biological behavior of MANs and improving diagnostic approaches. This case report presents a 34-year-old woman with a borderline myxoid adrenocortical neoplasm (BMAN) of the left adrenal gland, highlighting the diagnostic complexity and the clinical significance of complete surgical excision.

## Case presentation

A 34-year-old female patient presented to the outpatient clinic of Attikon University Hospital with a three-month history of persistent headaches, paroxysmal hypertension, and fatigue. During physical examination, the patient’s blood pressure fluctuated between 140-200 and 90-110 mmHg on three repeated measurements. Her heart rate was 82 beats/minute, with a regular ECG and cardiac rhythm. No Cushingoid features, abdominal bruits, or palpable masses were detected. The patient’s body mass index was calculated to be 24.5 kg/m², which is considered normal. No significant past medical history or family history of hypertension or cardiovascular disease was reported.

Laboratory studies revealed elevated plasma normetanephrine (116 ng/dL; normal values: 11.0-21.0 ng/dL) and norepinephrine (788 pg/mL; normal values: 70-1,700 pg/mL) secretion. However, the remaining hormonal profile did not reveal any significant changes.

Imaging with an abdominal CT scan revealed an incidental, nodular lesion at the lateral margin of the left adrenal gland, measuring 1.6 cm in diameter, with a pre-contrast density of 39 HU, and a post-contrast enhancement density of 120 HU. An MIBG scan was negative for metastatic or hypermetabolic lesions. Renal Doppler excluded renal artery stenosis.

The patient underwent preoperative hormonal blockade for two weeks, with an alpha antagonist (doxazosin) and calcium channel blockers. Subsequently, posterior retroperitoneoscopic adrenalectomy of the left adrenal gland was performed successfully.

**Figure 1:**
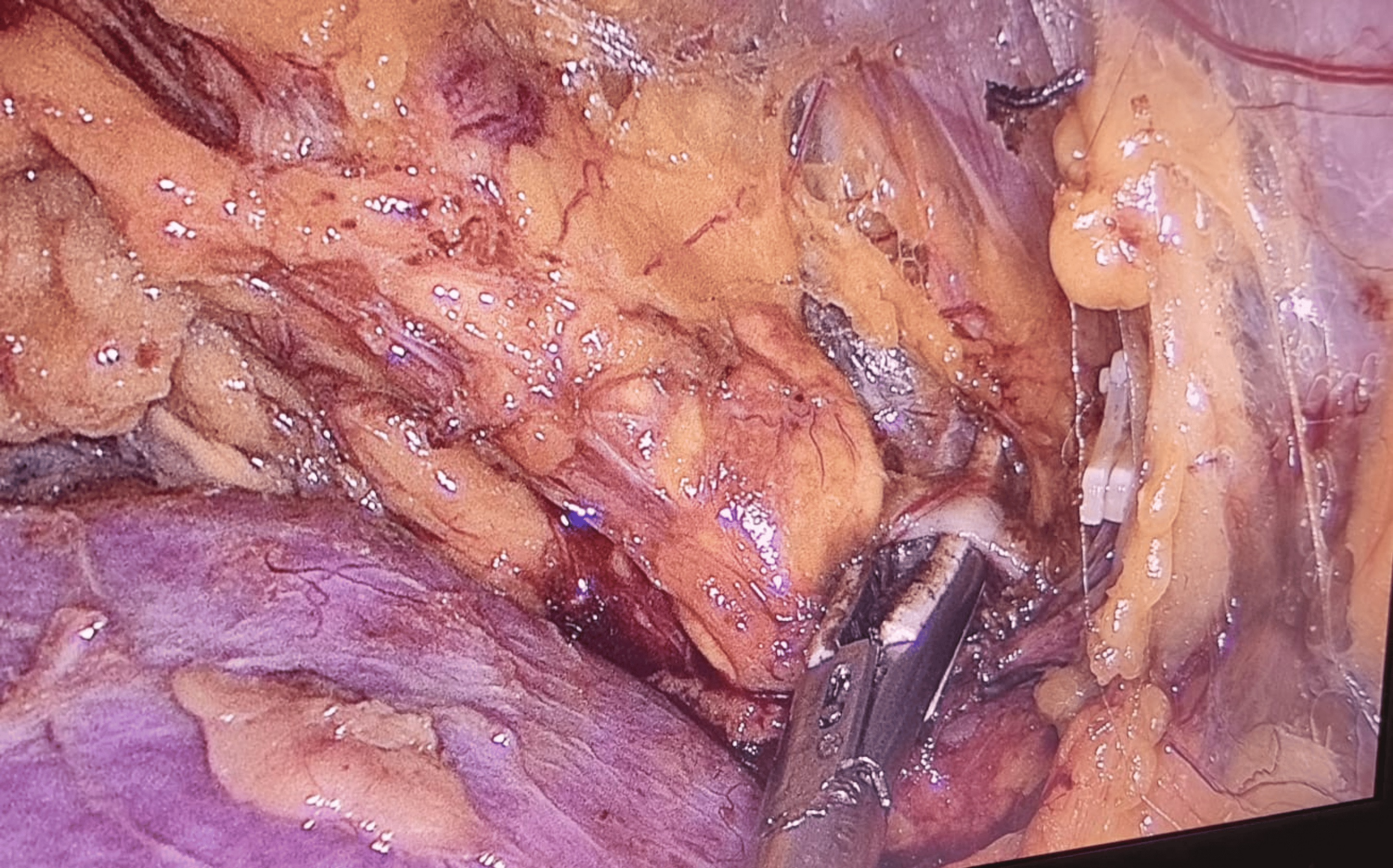
The left adrenal vein is identified after careful dissection.

**Figure 2:**
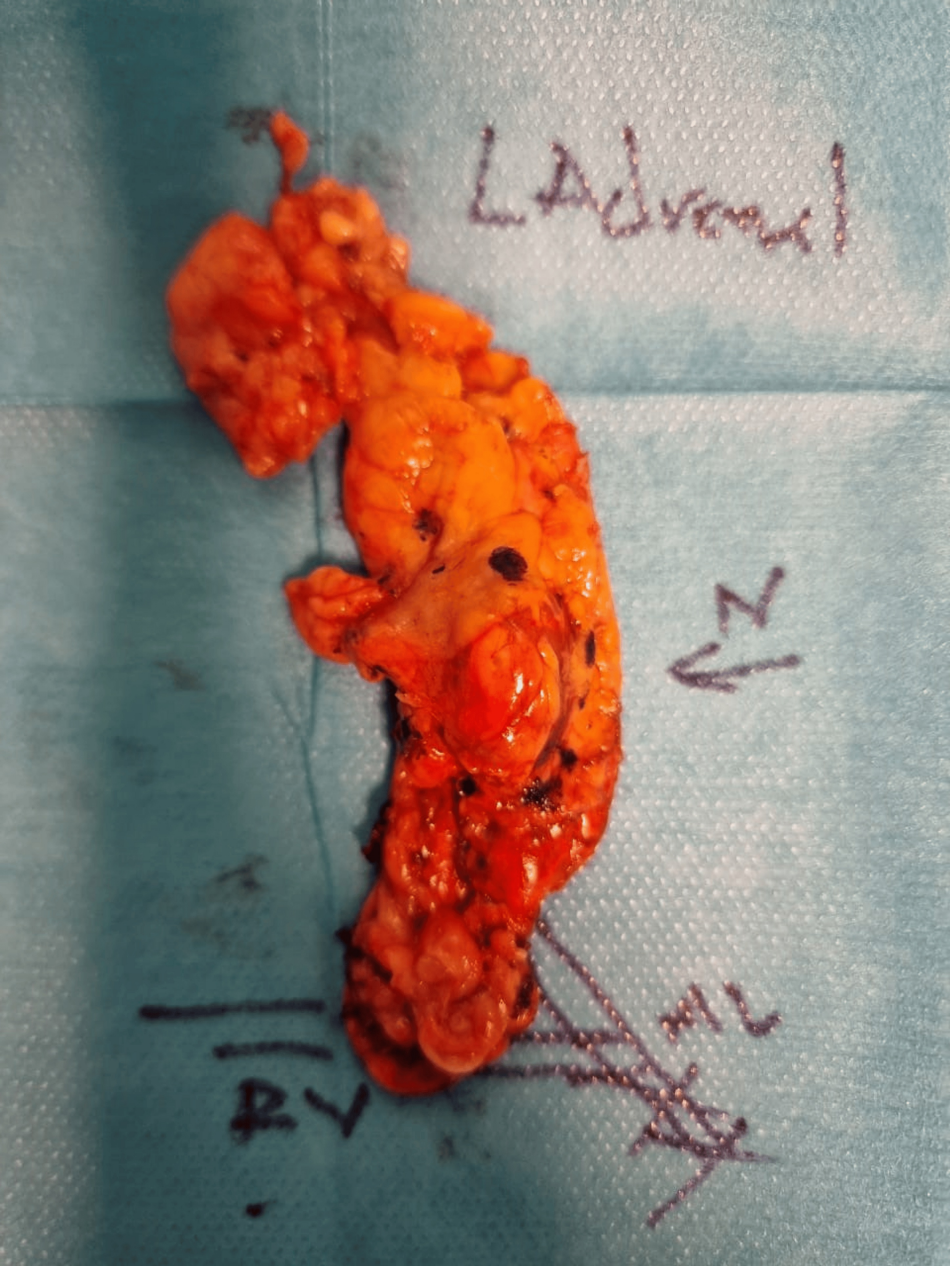
Left adrenal specimen presented with a drawing showing the vessels and the position of the tumor.

The patient was placed in the supine position, and the procedure was performed as described by Walz et al. [8]. The first incision was performed above the tip of the 12th left rib, and through that a 10 mm trocar was inserted. The other two trocars, 10 mm and 5 mm, were placed under finger protection in the space created within Gerota’s fascia through sharp dissection. Insufflation of CO_2_ was started and maintained at a high pressure of 25 mmHg. Retroperitoneoscopy was performed, and the dissection began using ultrasonic scissors. The adrenal gland was dissected from the upper pole of the kidney after its identification. Subsequently, the left adrenal vein was located and ligated (Figure 1). Following the complete excision of the adrenal gland, the specimen was removed with a retrieval bag (Figure 2).

**Figure 3:**
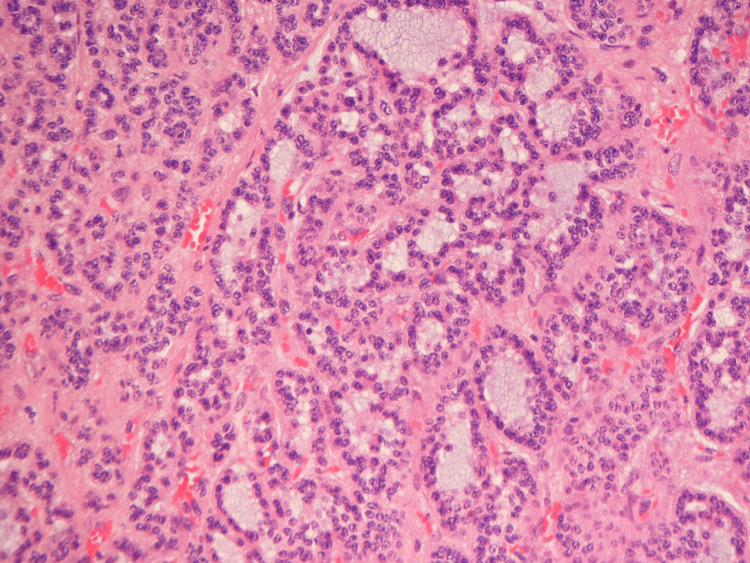
Hematoxylin and eosin staining (total magnification ×400).

The resected specimen was submitted for histopathological analysis. A 1.7 cm adrenal cortex tumor was identified. On hematoxylin and eosin staining, the tumor exhibited prominent myxoid features and was composed of flattened to cuboidal cells with lobulated nuclei and nuclear indentations (Figure 3). The architectural pattern included trabecular, pseudotubular, and pseudo-cribriform arrangements. No necrosis or mitotic activity was observed. There was no evidence of capsular, vascular, or periadrenal adipose tissue invasion.

**Figure 4:**
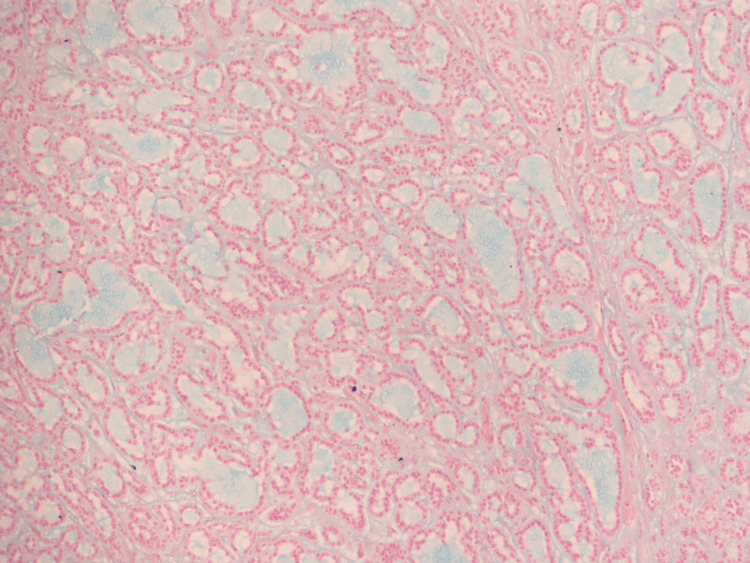
Alcian blue staining (total magnification ×100).

**Figure 5:**
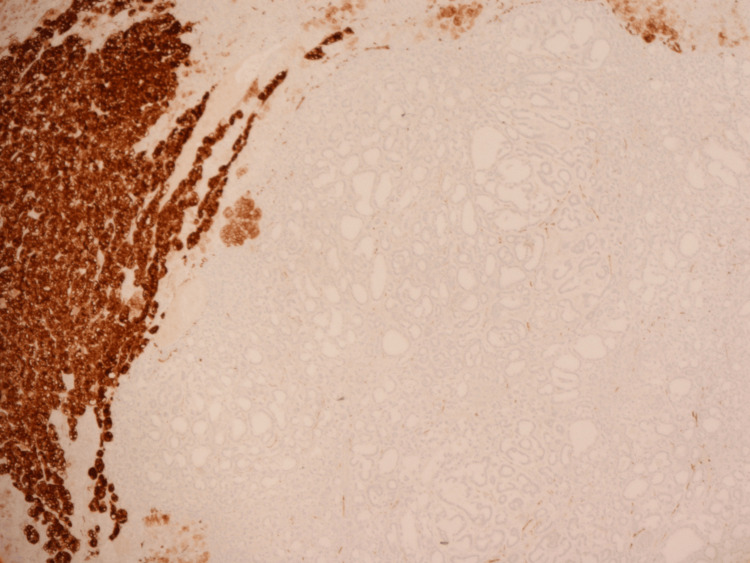
Synaptophysin immunohistochemical staining (total magnification ×40).

**Figure 6:**
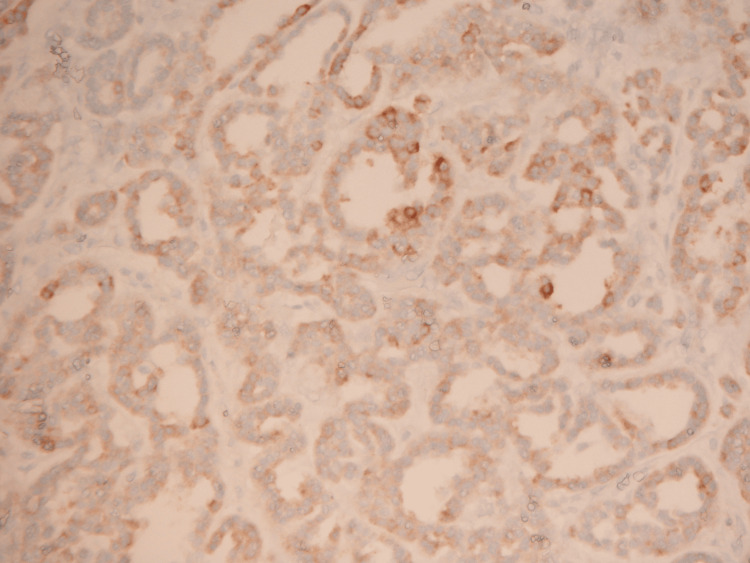
Inhibin A immunohistochemical staining (total magnification ×100).

**Figure 7:**
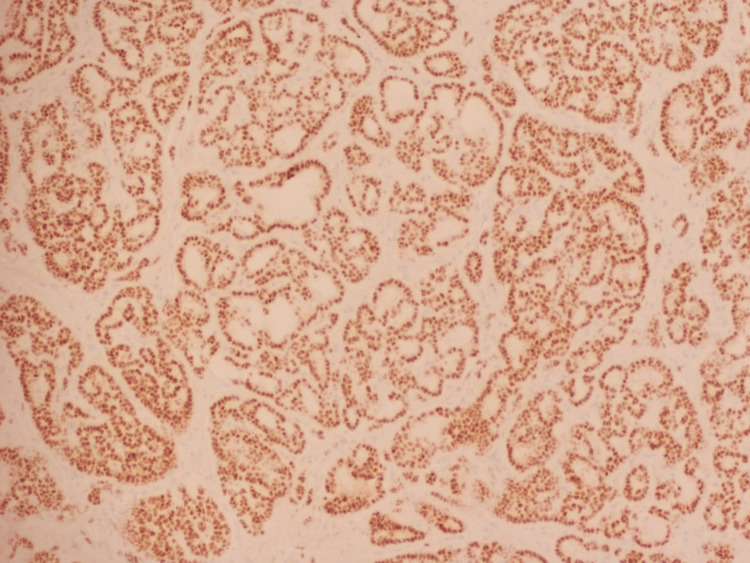
Steroidogenic factor 1 immunohistochemical staining (total magnification ×200).

**Figure 8:**
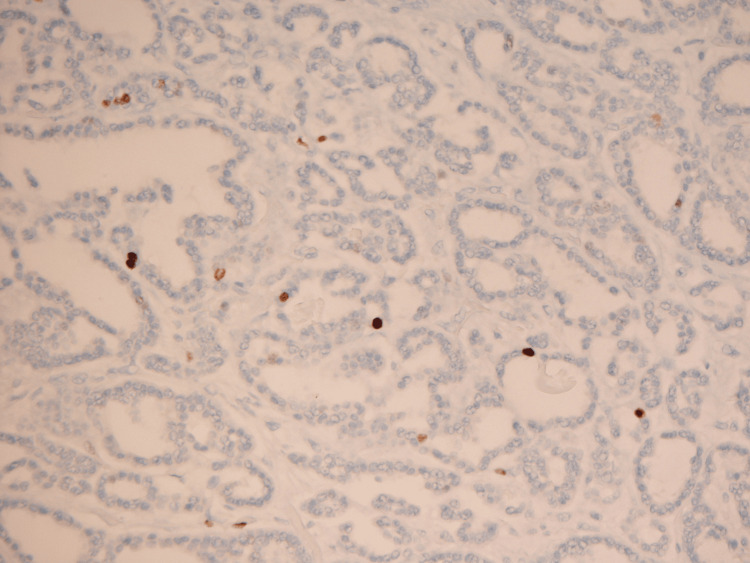
Ki-67 staining (total magnification ×100).

Histochemical staining was positive for Alcian blue (Figure 4), while periodic acid-Schiff and mucicarmine stains were negative. Immunohistochemical analysis demonstrated positivity for synaptophysin, inhibin A, and steroidogenic factor 1 (SF-1) (Figures 5-7). The Ki-67 proliferation index was low (0.8%) (Figure 8). Based on these findings, the Weiss score was calculated as 2. Overall, these features were consistent with a diagnosis of a BMAN.

The postoperative course was uneventful. During a three-month follow-up period, the patient’s blood pressure and catecholamine levels normalized, and she remained asymptomatic. After one year, the follow-up with hormonal evaluation and CT scan was negative.

## Discussion

MANs represent a rare histological variant of adrenal tumors, first described by Tang et al. in 1979 [4]. Since then, fewer than 100 cases have been reported [2,5,6]. The case of our 34-year-old female patient is remarkable due to the neoplasm’s borderline biological behavior. This category underscores a diagnostic challenge due to indefinite boundaries between adrenocortical adenomas and carcinomas [1,2,3].

The main characteristic of MANs is the extracellular myxoid matrix, which can constitute between 10% and 95% of the tumor volume [1,7,9]. Histologically, the cells in these tumors may be arranged in branching cords, microcysts, or even pseudoglandular patterns [7,10]. The pseudoglandular pattern, also observed in our case, may indicate a diagnostic difficulty, as it can resemble metastatic adenocarcinoma [10].

Histopathological assessment and management of MANs is particularly demanding. The Weiss score remains the gold standard method for histopathologic assessment and malignancy risk stratification in conventional adrenocortical tumors (Tables 1, 2); however, its applicability is limited in rare variants, particularly myxoid, oncocytic, and sarcomatoid subtypes [3,10]. In these subtypes, classical markers of malignancy such as atypia, diffuse growth, mitotic figures, invasion, or necrosis may be less evident or distorted by the extensive myxoid stroma [4,9,10]. Additionally, according to a few reports, some MANs may exhibit aggressive clinical behavior even when they present with borderline Weiss scores of 1 or 2, as observed in our patient [2,7,9]. Consequently, some studies recommend that the presence of a substantial myxoid component itself may be considered a sign of potentially unfavorable outcomes [7,11].

**Table 1:** Weiss score. A nine-parameter system used to differentiate adenomas and carcinomas. Each parameter scores 1 if present. Tumors exhibiting ≥3 positive criteria are classified as malignant. Those with <3 are considered adenomas. When the score is 1 or 2 and atypical or indeterminate morphological features are present, the tumor is categorized as having borderline malignant potential [10].

Morphological features evaluated
1. High nuclear grade (3 and 4)
2. Mitotic rate >5/50 HPF
3. Atypical mitoses
4. Eosinophilic cytoplasm in >75% of cells
5. Diffuse architecture (>33% tumor)
6. Tumor necrosis
7. Venous invasion
8. Sinusoidal invasion
9. Capsular invasion

**Table 2:** Modified Weiss score. A simplified version focusing on more objective parameters (commonly 5). The cutoff for malignant behavior is ≥3 [10].

Morphological features evaluated
1. Mitotic rate >5/50 HPF
2. Clear cells ≤25%
3. Atypical mitoses
4. Tumor necrosis
5. Capsular invasion

Considering the limited utility of the Weiss score in myxoid variants, recent literature recommends the application of complementary scoring systems [9,11]. A more objective alternative is the reticulin algorithm (Table 3). It focuses on the disruption of the reticulin framework, which appears to be a more reliable indicator of malignancy in tumors with myxoid stroma [9,12,13]. Additionally, the Helsinki scoring system (Table 4) has been suggested for improved risk stratification and incorporates mitotic rate, presence of necrosis, and the Ki-67 proliferation index [11,14]. In our patient, despite the estimated borderline Weiss score (2) and extensive myxoid change, the low Ki-67 index (often <5 in borderline cases) and the absence of reticulin framework disruption supported the diagnosis of a BMAN rather than a carcinoma [11,14]. This holistic approach is aligned with the 2022 WHO Classification guidelines, which recommended the evaluation of histologic, immunohistochemical, and molecular findings [3]. This methodology is crucial when encountering a tumor with predominant myxoid stroma due to the risk for under-recognition, delayed diagnosis, or misclassification [3,9].

**Table 3:** Reticulin algorithm. Diagnosis of carcinoma requires an altered reticulin framework and at least one of necrosis, high mitotic rate, or vascular invasion [13].

Morphological features evaluated
1. Altered/Absent reticulin framework
2. Tumor necrosis (coagulative necrosis)
3. High mitotic rate (>5/50 HPF)
4. Vascular invasion

**Table 4:** Helsinki scoring system. The cutoff for malignancy is >8.5. Scores >8.5 are highly predictive of metastatic behavior and malignancy [14].

Feature	Scoring contribution
1. Mitotic rate >5/50 HPF	×3
2. Tumor necrosis present	×5
3. Ki-67 index (%)	+ % value

Immunohistochemistry is fundamental for the confirmation of the adrenocortical origin of MANs, as well as differentiation from other retroperitoneal myxoid tumors [10,11]. These are myxoid liposarcoma, myxoid leiomyosarcoma, neuroendocrine tumors, sex cord-stromal tumors, and metastatic carcinomas from the pancreas or the gastrointestinal tract [7,9]. As indicated in the literature, MANs typically show positive expression for vimentin, synaptophysin, alpha-inhibin, melan-A, and SF-1 and negative expression for cytokeratin [7,9-12].

MANs are usually asymptomatic and detected incidentally. However, a minority of cases may exhibit mild or subclinical hormone excess, occasionally leading to electrolyte disturbances such as hypokalaemia [2,4,5]. In our case, the patient’s presentation was atypical, with episodic hypertension and elevated catecholamine levels, which initially raised suspicion for a pheochromocytoma. Nonetheless, a negative MIBG scan, combined with histopathological evaluation, confirmed the myxoid adrenocortical origin.

On radiological imaging, MANs lack specific features that reliably differentiate them from conventional adrenocortical neoplasms [2,5]. CT and MRI may reveal well-defined lesions with signal intensity variations consistent with the mucin content; however, definitive diagnosis remains exclusively histopathological [2,5].

The prognosis for MAN is generally considered more aggressive than for conventional adrenocortical carcinoma. There is a higher propensity for early metastasis and reduced survival rates in malignant cases [11,12]. However, in benign adenomas and borderline lesions (BMAN), complete surgical removal, as performed in this case via retroperitoneoscopic adrenalectomy, is typically sufficient for cure [1,10]. Additional treatments, including the adrenolytic antineoplastic agent mitotane, chemotherapy [15], and developing targeted therapy using tyrosine kinase inhibitors targeting epidermal growth factor receptor and other pathways, may be added, particularly in tumors with increased malignancy potential [15,16].

Nonetheless, the diagnostic uncertainty and the potential for aggressive behavior even in borderline lesions necessitate long-term follow-up [11,12]. Owing to the rarity of MANs, there are no specific evidence-based follow-up guidelines. Therefore, surveillance is generally based on approaches used for adrenocortical carcinomas and tumors of uncertain malignant potential, with our patient undergoing follow-up at three months and one year [17,18]. Although the postoperative normalization of our patient’s blood pressure and catecholamine levels was a favorable prognostic sign, continuous surveillance remains crucial due to the limited knowledge of this rare histological variant.

## Conclusions

MANs represent a heterogeneous entity of adrenal cortical tumors. The differentiation between benign, borderline, and malignant neoplasms remains challenging. The present case illustrates the importance of a comprehensive diagnostic approach in evaluating these neoplasms. Histopathological evaluation remains fundamental in establishing the final diagnosis, considering the absence of specific imaging features. Surgical excision remains the treatment’s foundation and is curative in benign and BMANs, as indicated by the favorable outcome in our patient. Additional cases with detailed clinicopathologic correlation will contribute to a better understanding of the biological behavior of this variant, refine diagnostic criteria, and improve risk stratification.
